# *Campylobacter coli* Clade 3 Isolates Induce Rapid Cell Death *In Vitro*

**DOI:** 10.1128/AEM.02993-18

**Published:** 2019-02-20

**Authors:** Cecilia Johansson, Anna Nilsson, René Kaden, Hilpi Rautelin

**Affiliations:** a Clinical Microbiology, Department of Medical Sciences, Uppsala University, Uppsala, Sweden; INRS—Institut Armand-Frappier

**Keywords:** *Campylobacter coli*, IL-8, cell necrosis, clade 1, clade 2, clade 3, gene expression, *in vitro* infection, phylogenetic analysis, virulence

## Abstract

*Campylobacter* bacteria are major human enteropathogens. Campylobacter coli shows less genetic diversity than C. jejuni and clusters into three clades, of which clade 1 includes most human and farm animal isolates, while environmental C. coli isolates mainly belong to clades 2 and 3. Recently, we sequenced the whole genomes of eight C. coli clade 2 and 3 isolates cultivated from water, and here we studied their interaction with human HT-29 colon cancer cells compared to that of clinical clade 1 isolates. All C. coli clade 3 isolates already caused cell necrosis 1 to 2 h after inoculation, whereas none of the clade 1 and 2 isolates analyzed induced cell death. Isolates from clades 2 and 3 adhered to epithelial cells better than clade 1 isolates, but all isolates induced similar levels of interleukin-8 (IL-8). Alignment and phylogenetic analysis of the translated putative virulence genes *cadF*, *flpA*, *iamA*, *ciaB*, and *ceuE* revealed clade-specific protein sequence variations, with clade 1 and 2 sequences being more closely related and clade 3 sequences being further apart, in general. Moreover, when RNA levels were measured, clade 3 isolates showed significantly lower levels of expression of *cadF*, *iamA*, and *ceuE* than clade 2 isolates, while *flpA* expression levels were higher in clade 3 isolates. The cytolethal distending toxin genes were also expressed in clades 2 and 3, although there was no difference between clades. Our findings demonstrate differences between the effects of C. coli clade 1, 2, and 3 isolates on human cells and suggest that C. coli clade 3 might be more virulent than clade 2 due to the observed cytotoxicity.

**IMPORTANCE** Campylobacter coli is a common zoonotic cause of gastroenteritis in humans worldwide. The majority of infections are caused by C. coli clade 1 isolates, whereas infections due to clade 2 and 3 isolates are rare. Whether this depends on a low prevalence of clade 2 and 3 isolates in reservoirs important for human infections or their lower ability to cause human disease is unknown. Here, we studied the effects of C. coli clade 2 and 3 isolates on a human cell line. These isolates adhered to human cells to a higher degree than clinical clade 1 isolates. Furthermore, we could show that C. coli clade 3 isolates rapidly induced cell death, suggesting differences in the virulence of C. coli. The exact mechanism of cell death remains to be revealed, but selected genes showed interesting clade-specific expression patterns.

## Abstract (precis)

Campylobacter coli is a common zoonotic cause of gastroenteritis in humans worldwide. The majority of infections are caused by C. coli clade 1 isolates, whereas infections due to clade 2 and 3 isolates are rare. Whether this depends on a low prevalence of clade 2 and 3 isolates in reservoirs important for human infections or their lower ability to cause human disease is unknown. Here, we studied the effects of C. coli clade 2 and 3 isolates on a human cell line. These isolates adhered to human cells to a higher degree than clinical clade 1 isolates. Furthermore, we could show that C. coli clade 3 isolates rapidly induced cell death, suggesting differences in the virulence of C. coli. The exact mechanism of cell death remains to be revealed, but selected genes showed interesting clade-specific expression patterns.

## INTRODUCTION

*Campylobacter* bacteria colonize the gastrointestinal tract of warm-blooded animals, such as poultry and wild birds, ruminants, pigs, cats, and dogs, but rarely cause any symptoms in animal hosts. However, when transmitted to humans, Campylobacter coli and C. jejuni in particular cause gastroenteritis worldwide (1). Indeed, *Campylobacter* has been the most commonly reported bacterial enteropathogen since 2005 in the European Union and has shown a continuous increasing trend since 2008 (2). It has even been estimated that about 1% of European inhabitants develop campylobacteriosis every year (3). The majority of *Campylobacter* infections have been attributed to the poultry reservoir as a whole (4), but the contribution of sources such as water seems to be important as well and warrants more research (5–8).

*Campylobacter* infection usually develops 1 to 5 days after ingestion of bacteria, with typical symptoms including fever, headache, vomiting, and abdominal pain, in addition to watery or, sometimes, bloody diarrhea (9). Following the acute diarrheal illness, *Campylobacter* patients might even develop postinfectious sequelae, such as reactive arthritis (10), irritable bowel syndrome (11), and the severe autoimmune demyelinating neuropathy Guillain-Barré syndrome (12). These complications substantially increase the burden of the disease, which in the United States has recently been estimated to be 22,500 disability-adjusted life years (13).

C. coli is less common than C. jejuni as a human pathogen and seems to account for about 10% of *Campylobacter* infections (14), although higher incidences have also been reported (15). In general, the clinical picture of C. coli infection cannot be distinguished from that of C. jejuni infection (14). In contrast to C. jejuni, C. coli isolates show less genetic diversity and cluster into three clades (16, 17). C. coli clade 1 includes two clonal complexes (CCs) and the majority of clinical and farm animal isolates (18), whereas clade 2 and clade 3 lack a CC structure and mainly consist of isolates from environmental origins (16). It is not known whether the low prevalence of C. coli clades 2 and 3 among human fecal isolates is merely due to the less common presence of these types of isolates in reservoirs important for human infections or if clade 2 and 3 isolates actually are less capable than clade 1 isolates of causing human infection.

The pathogenesis of *Campylobacter* infection is still largely unknown, although several virulence factors have been described (19). In general, campylobacteriosis is considered a multifactorial process in which bacterial motility, adhesion, and invasion, among several other virulence properties, seem to be important. CadF (*cj1478c*) and FlpA (*cj1279c*) are bacterial adhesins known to bind fibronectin on the host cell surface, an action which then initiates the invasion process (20–23). Invasion has been shown to thereafter be activated by secreted *Campylobacter* invasion antigens, Cia proteins. Although CiaB (*cj0914c*) has been shown to be needed for efficient invasion (24, 25), a more recent report has demonstrated a negative correlation between CiaB and invasiveness (26). Another possibly important factor is the invasion-associated marker, IamA (*cj1647*). Although its function is still unknown, it has been reported to be expressed in the majority of invasive *Campylobacter* isolates but only in a minority of noninvasive *Campylobacter* isolates (27).

In addition, the cytolethal distending toxin (CDT) is well-known among *Campylobacter* isolates and is also produced by many other bacterial pathogens (reviewed in reference 28). CDT consists of three subunits: the enzymatically active B subunit (*cj0078c*) and the A (*cj0079c*) and C (*cj0077c*) subunits, which assemble the active holotoxin, which binds to the cell membranes of host cells. The DNase activity of CdtB induces double-strand breaks in the host chromosomes, eventually leading to cell cycle arrest at the G_2_/M interphase. Moreover, CDT has been shown to induce interleukin-8 (IL-8) secretion from INT-407 cells through its binding to host cell membranes (29), possibly driving the inflammation in the intestine. Furthermore, hemolytic proteins, such as the lipoprotein CeuE (*cj1355*), which is also involved in iron release and uptake from the environment (30, 31), have been listed among the candidate *Campylobacter* virulence factors.

In addition to analyses of putative virulence factors, the bacterial pathogenic potential can be investigated using *in vitro* infection models showing interactions between bacteria and epithelial cell lines. In these assays, adherence to human cells (32), as well as the resulting inflammation due to the release of proinflammatory chemokines, like IL-8 (33, 34), can be quantified *in vitro*. We have earlier used INT-407 cells in such an infection model to compare the *in vitro* host responses of various clinical C. jejuni isolates and detected clear differences (35). However, as most research has focused on C. jejuni, such *in vitro* infection models analyzing C. coli isolates are quite few (36, 37). Furthermore, to the best of our knowledge, the effect of C. coli clade 2 and 3 isolates on human cells has not been described, until now.

We recently characterized eight C. coli water isolates originally cultivated from incoming surface water at water plants. These particular C. coli clade 2 and clade 3 water isolates showed clade-specific variation in gene content and phenotypic traits (38). In the work described here, we studied the interactions between these environmental C. coli isolates together with some clinical C. coli clade 1 isolates and human cells in an *in vitro* infection model using HT-29 colon epithelial cells.

(Parts of this study were presented as a poster at the 6th One Health Sweden Scientific Meeting A World in Transition—Changes in Infection Ecology, 17 to 18 March 2016, Uppsala, Sweden [39]; as a poster at the 19th International Workshop on Campylobacter, Helicobacter and Related Organisms, 10 to 14 September 2017, Nantes, France [40]; and as parts of Anna Nilsson’s Ph.D. thesis [41].)

## RESULTS

### C. coli clade 3 isolates are cytotoxic to HT-29 cells.

**FIG 1:**
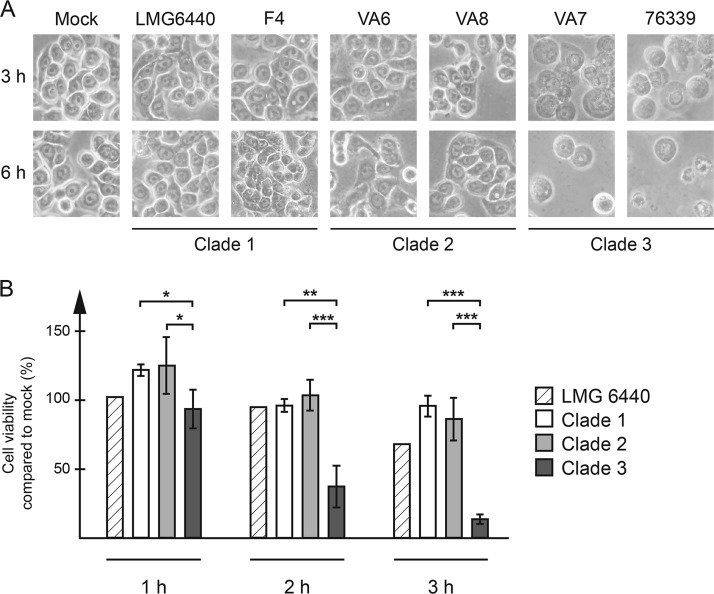
HT-29 cells were infected with each C. coli isolate at an MOI of 100 for the indicated time periods. (A) Representative images of infected cells at 3 and 6 h postinfection. (B) Extracellular lactate dehydrogenase activity was measured in the cell culture medium and expressed as the relative fraction of healthy cells, with the value for mock-infected cells being set to 100% for each time point. Data from three independent infection experiments were used. The graph shows the mean and standard deviation for the isolates in each clade (clade 1, *n* = 3 isolates; clade 2, *n* = 5 isolates; clade 3, *n* = 4 isolates). Significant clade-specific differences are indicated. *, *P* < 0.05; **, *P* < 0.01; ***, *P* < 0.001.

Using our previously published (42, 43) *in vitro* infection model with human HT-29 colon cancer cells, all clade 3 isolates tested had a strong cytotoxic effect on the cells. The cells showed clear signs of cell necrosis, with swelling, membrane disruption, and, finally, cell lysis already being seen 1 to 2 h after inoculation of bacteria into the cells (Fig. 1A). The water isolate VA7 and the clinical isolate 76339 had the strongest effect on the cells, with the cells already showing clear signs of necrosis after 1 h, while water isolates VA15 and VA38 had a slightly delayed effect (data not shown). All of these C. coli clade 3 isolates also had the same effect on Caco-2 and HeLa cells (data not shown). The cytotoxic effect was dependent on viable bacteria, as heat-inactivated bacteria or broth from overnight cultures had no such effect (data not shown). Neither the C. coli clade 1 and 2 isolates nor C. jejuni reference strains 11168 and 81-176, showed the same cytotoxic effect (Fig. 1A and data not shown).

In order to quantify the toxic effect, lactate dehydrogenase (LDH) activity, an indicator for reduced cell viability, from ruptured cells was measured in the cell culture media at different time points after infection (Fig. 1B). C. coli clade 3-infected cells showed significantly reduced viability already at 1 h postinfection and only 13% viability at 3 h, while C. coli clade 1- and 2-infected cells remained viable (Fig. 1B).

### C. coli water isolates adhere to HT-29 cells and induce an IL-8 response.

**FIG 2:**
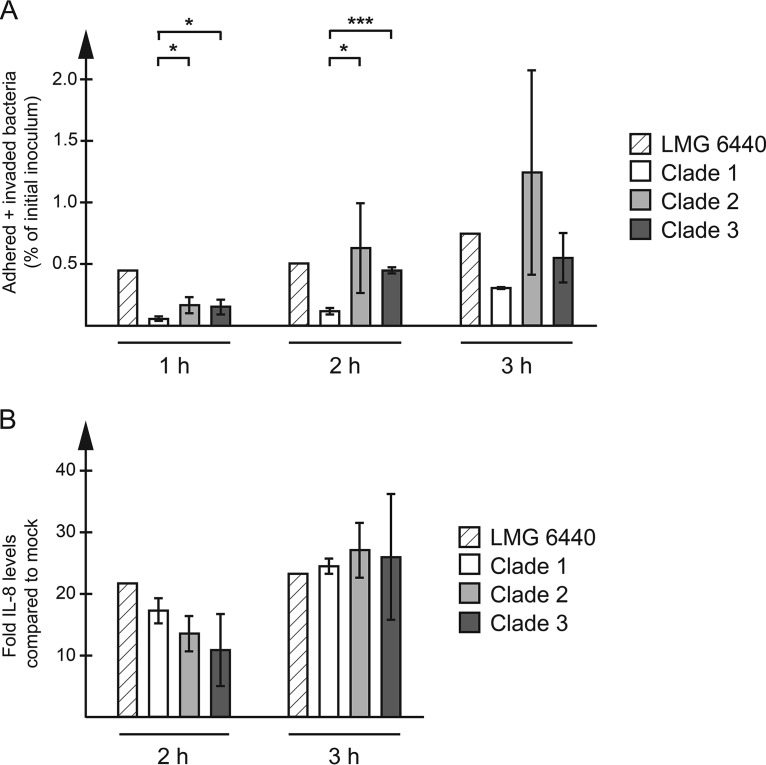
HT-29 cells were infected with each C. coli isolate at an MOI of 100 for the indicated time periods. (A) Cells were harvested and lysed, and adhered bacteria were quantified using qPCR, with the amount being expressed as a percentage of the starting inoculum. Data from three independent infection experiments were used. The graph shows the mean and standard deviation for the isolates in each clade (clade 1, *n* = 3; clade 2, *n* = 5; clade 3, *n* = 4). Significant clade-specific differences are indicated. *, *P* < 0.05; ***, *P* < 0.001. (B) IL-8 levels in the cell culture medium were measured using ELISA and expressed as the fold increase over that for mock-infected cells. The graph shows the mean and standard deviation for the isolates in each clade (clade 1, *n* = 3; clade 2, *n* = 5; clade 3, *n* = 4).

To see whether the demonstrated cytotoxicity correlated with the ability of the isolates to adhere to host cells, bacteria were harvested from the cells and quantified using quantitative PCR (qPCR). All C. coli water isolates were able to adhere to HT-29 cells (Fig. 2A), and adhesion levels increased with time (Fig. 2A) to between 0.4 and 2.4% at 3 h postinfection (Fig. 2A). The individual clade 2 isolates varied substantially in their ability to adhere at 2 and 3 h postinfection, with the levels at 3 h being 2.4% for VA6, 0.89% for VA8, 1.8% for VA24, 0.48% for VA37, and 0.64% for VA46 (Fig. 2A and data not shown). The clade 2 and 3 water isolates adhered significantly better than the clade 1 isolates at the 1- and 2-h time points (Fig. 2A). For clade 1 isolates, the adherence was in the same range as that for the C. jejuni reference strains 11168 and 81-176 (about 0.4%; data not shown). There was no significant difference between clade 2 and clade 3 isolates at any time point (Fig. 2A).

All C. coli isolates could elicit an IL-8 response (Fig. 2B), which was detectable in the cell culture media 2 h after inoculation and which was also in the same range as that elicited by C. jejuni 11168 and 81-176 (data not shown). The levels of IL-8 elicited by clade 3 clinical isolate 76339 were lower than those elicited by all other isolates at both time points (data not shown). However, there were no significant clade-specific differences at any time point (Fig. 2B).

### Clade-specific sequence variations in putative virulence genes.

The whole genomes of clade 1 isolates F3, F4, and F8 and the LMG 6440 reference strain were sequenced and, together with the whole-genome sequences of previously sequenced clade 2 and 3 isolates (38, 44) and the whole-genome sequences of an additional 78 C. coli clade 2 and 3 isolates that were retrieved from the NCBI database, used for *in silico* analyses of genes and proteins. The clade assignments of all selected isolates were confirmed by phylogenetic analyses of the whole-genome sequences (data not shown).

We analyzed the genetic structure of selected virulence genes, namely, *cadF*, *flpA*, *iamA*, *ciaB*, and *ceuE*. The sequence alignments of each gene from all isolates in Table S1 in the supplemental material revealed that the particular genes studied had nucleotide sequence identities ranging from 83.6 to 89.5% (Table 1). Gene open reading frames (ORFs) were further translated *in silico*, which revealed protein sequence identities of 87.3 to 97.0% (Table 1).

**TABLE 1:** DNA and amino acid sequence identities of five putative virulence genes between C. coli clade 1, 2, and 3 isolates as assessed *in silico*^*a*^

Gene	Sequence identity (%)	
	Nucleotide	Amino acid
*cadF*	83.6	87.3
*flpA*	85.0	89.0
*iamA*	88.4	95.4
*ciaB*	85.1	90.2
*ceuE*	89.5	97.0

a Data are for 91 isolates (see Table S1 in the supplemental material). Numbers show the percentage of sites with identical nucleotides or amino acids upon alignment of all sequences.

**FIG 3:**
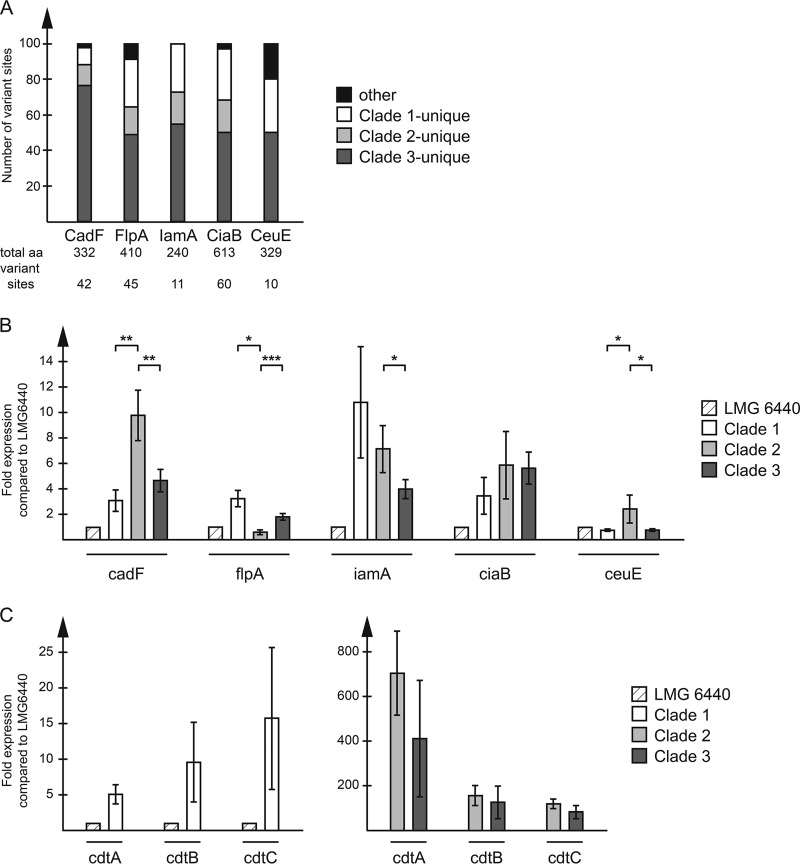
(A) Distribution of amino acid variations in selected putative virulence proteins. Stacked bars show the percentage of sites with unique clade 1, unique clade 2, unique clade 3, and other types of variations. The numbers below each bar show the total number of amino acids (aa) and the total number of variant sites in each protein. (B, C) The expression of putative virulence genes was analyzed using RT-qPCR. Data are expressed as the value relative to that for the clade 1 reference strain LMG 6440 for each gene and shown as the mean and standard deviation for the isolates in each clade (clade 1, *n* = 3; clade 2, *n* = 5; clade 3, *n* = 4). Significant clade-specific differences are indicated. *, *P* < 0.05; **, *P* < 0.01; ***, *P* < 0.001.

Alignment and phylogenetic analyses of all protein sequences of the selected virulence genes showed that sequence variations were highly conserved within each clade, and each protein sequence alignment yielded phylogenetic trees with strict clade divisions for all virulence genes analyzed (Fig. S1 and S3 and data not shown). Furthermore, for all genes analyzed, the clade 3 unique variations, i.e., where the clade 3 sequence differed from the sequences of clades 1 and 2, were clearly outnumbered by all other combinations (Fig. 3A).

For CadF, 76% (32 out of 42) of the amino acid variations were unique to clade 3 (Fig. 3A and S2). The most striking difference was the lack of an 18-nucleotide-long region in the middle of the clade 3 *cadF* gene, resulting in a 6-amino-acid gap, a feature also found in the C. jejuni reference strains studied. This gap was located within a peptidoglycan-binding OmpA-like domain (spanning amino acids 167 to 324), which is also where most of the additional sequence variations in the CadF protein were found (Fig. S2). Of the 32 variant amino acids in CadF unique to clade 3, 16 were identical to those of the C. jejuni strains (Fig. S2), and the phylogenetic tree (Fig. S1) also confirmed that C. jejuni CadF was more closely related to the CadF of C. coli clade 3 than to that of clades 1 and 2. For CeuE, the phylogenetic tree showed an even closer relationship between C. jejuni and C. coli clade 3 (Fig. S3).

### Differences in expression levels of putative virulence genes.

In order to assess whether the detected virulence genes were actually expressed in our C. coli isolates at the time of infection, RNA levels were measured in overnight cultures using reverse transcription (RT)-qPCR. The clade 3 isolates had significantly lower expression levels of the *cadF*, *iamA*, and *ceuE* genes than the clade 2 isolates but showed significantly higher expression levels of the *flpA* gene (Fig. 3B). There was no significant difference in the expression levels of *ciaB* between clades 1, 2, and 3 (Fig. 3B). Moreover, clade 2 and 3 isolates had very high expression levels of the *cdtA*, *cdtB*, and *cdtC* genes compared to the clade 1 isolates, with the expression levels for the clade 2 and 3 isolates ranging from being 10- and 50-fold over those for the clade 1 isolates (Fig. 3C).

### Analysis of clade 3-specific genes.

Selected clade 3-specific genes, earlier identified by us (38), were further analyzed here. A putative secreted serine protease gene, specific to all our clade 3 water isolates and also found in the clinical clade 3 isolate 76339, was also detected in all other clade 3 sequences included in our sequence data set (Table S1) but was not found in any clade 1 or 2 sequences studied here. BLASTp analysis of the translated ORFs identified this particular protein in many other C. coli isolates (clades unknown) with identities of 90 to 99%.

Among the other earlier identified clade 3-specific genes (38), the presence of genes coding for eight hypothetical proteins was also studied. These genes were translated here for identification of possible functions. Only two of these particular genes were present in all clade 3 sequences studied here and had intact open reading frames. These two were further identified by BLASTp analysis as a hypothetical protein of the DUF1090 superfamily and a C. coli metallo-dependent amidohydrolase with 77% similarity to the C. jejuni counterpart; the functions of both ORFs are unknown. Furthermore, the presence of the putative secreted serine protease and the amidohydrolase genes in only the clade 3 sequences was further verified using qPCR and RNA expression analyses of both these genes, which showed similar expression levels in all clade 3 isolates, while they were undetected in the clade 2 isolates (data not shown).

## DISCUSSION

We recently used comparative genomics and phenotypic analyses and revealed several clade-specific differences between C. coli clade 2 and 3 water isolates (38). As C. coli clade 2 and 3 isolates, in contrast to those of clade 1, have only rarely been detected in human infections, we wanted to study if the clade-specific differences detected in our C. coli water isolates could actually be reflected in the ability of these particular isolates to infect human cells. For this purpose, we used a previously established *in vitro* infection model of human HT-29 colon cancer cells (42, 43) and, to our surprise, could reveal striking differences between the clades.

In contrast to clade 1 and 2 isolates, clade 3 isolates had a strong visual cytotoxic effect on the cells with the corresponding release of LDH activity into the cell culture media, a phenomenon commonly seen with disrupted cell membranes, indicative of necrosis. This effect was already detected 1 to 2 h after inoculation of the cells. The fact that there were no signs of programmed cell death (apoptosis), such as cell shrinkage, membrane blebbing, and condensed chromatin, suggests that the cell death was triggered by an external factor, i.e., the binding of bacteria to the host cell or a secreted toxin.

When we assessed the adhesion levels of the C. coli isolates, we found no correlation between the cytotoxicity and the ability to adhere to the cells, as several of the nontoxic clade 2 isolates had higher levels of adhesion than the cytotoxic clade 3 isolates at the 2- and 3-h time points. However, for clade 2 isolates, the cells might have remained alive long enough to allow a more efficient adhesion. In addition, unexpectedly, the clade 2 and 3 water isolates showed significantly higher levels of adhesion than the clinical clade 1 isolates, but the significance of this finding remains to be studied. Furthermore, it remains to be studied if there are any differences in invasiveness between the isolates belonging to different clades, as in this study, we only looked at the combined, and not separate, levels of adhered and invaded bacteria.

In this study, we detected high frequencies of amino acid variations, unique to clade 3, in four virulence proteins earlier shown to be involved in adhesion and invasion (CadF, FlpA, IamA, and CiaB). CadF and FlpA are fibronectin-binding adhesion factors, shown to be involved in the initiation of *Campylobacter* invasion (20–23), while IamA and CiaB have been implicated to have a role later on in the invasion process (24, 25, 27). Phylogenetic and genetic analyses of translated amino acid sequences of the *cadF*, *flpA*, *iamA*, and *ciaB* genes revealed a closer relation between isolates of clades 1 and 2, with clade 3 isolates being further away, in general (see Fig. S1 in the supplemental material). It remains to be studied if these particular proteins contribute to the differences seen in adhesion levels.

The C. coli clade 3 CadF proteins showed a higher similarity to C. jejuni CadF proteins, in particular, the 6-amino-acid gap close to the fibronectin-binding site, than to those of C. coli clade 1 and 2 isolates. This difference in protein size has earlier been reported to lead to a higher INT-407 cell invasion by C. jejuni isolates than by C. coli clade 1 isolates, although isolates of both species showed similar protein levels (45). It is, however, possible that this effect is counteracted by the lower expression level of the *cadF* gene in clade 3 isolates than in clade 2 isolates, as suggested in the present study.

CiaB has been shown to promote invasion indirectly via the activation of cellular Rho GTPases inducing cell membrane ruffling (46, 47). In the present study, *ciaB* was expressed to the same level in all the isolates studied, but the possibility that the clade-specific differences in the protein sequence could affect the regulation of Rho GTPase signaling and, thereby, invasion cannot be excluded.

The *ceuE* gene showed higher expression levels in clade 2 than clades 1 and 3, probably excluding the possibility that its hemolytic activity is the direct cause of the cytotoxic effect. However, the high expression level of *ceuE* is likely an advantage for the survival of clade 2 bacteria in the environment (30, 31).

One possible explanation for the cytotoxic effect demonstrated in the present study could be that a secreted factor (toxin) directly triggers necrosis. Many bacterial pathogens use this strategy to block cellular functions to better avoid host defense and facilitate bacterial replication. CDT is well-known in *Campylobacter* and has also been reported to contribute to the inflammation by indirectly inducing IL-8 through disruption of cell cycle progression (29, 48). However, several findings here speak against a secreted toxin causing the clade 3-specific cytotoxic effect. First, broth from overnight bacterial cultures had no cytotoxic effect. This might indicate that the active binding of living bacteria is needed, a hypothesis further supported by the finding that heat-inactivated bacteria could not induce cytotoxicity. Second, the very fast cytotoxic effect of clade 3 isolates points more toward a more directly acting lytic function, as the amounts of secreted factors would be low in the newly diluted cultures used here for infection. Third, the expression levels of *cdtABC*, as well as the IL-8 levels, were similar in clade 2 and 3 isolates, probably ruling out CDT as the effector for clade 3-specific cytotoxicity.

It also remains to be investigated if any of the clade 3-specific genes identified earlier by us (38) is responsible for the cytotoxic effect. It is interesting that several clade 3-specific putative proteins seemed to be hydrolytic and involved in the enzymatic degradation of cellular components, as infection-induced necrosis of host cells is often caused by toxins with hydrolytic properties. One such protein of interest is the putative secreted serine protease, the ORF for which we could also identify in earlier published C. coli clade 3 sequences. Serine protease autotransporters have earlier been implicated in bacterial toxicity, and, for example, serine protease autotransporters of *Enterobacteriaceae* (SPATEs) are well characterized as toxins connected to pathogenicity (49). The Escherichia coli Pet toxin alters the cellular cytoskeleton, causing swelling and disruption of both Hep-2 and HT-29 cells, a phenomenon shown to be dependent on the serine protease domain (50, 51). This effect of E. coli strongly resembles that of C. coli clade 3 isolates on HT-29 cells in this study, making the clade 3-specific putative secreted serine protease identified here an interesting candidate for the clade 3-specific cytotoxicity. However, it remains to be studied in more detail whether this particular protein plays a part in the clade 3 cytotoxicity shown here or in the virulence of C. coli clade 3 in general.

This study shows that *Campylobacter* can induce rapid cell death. Although the exact mechanism and the possible involvement of the clade 3-specific serine protease for cytotoxicity still remain to be revealed, these results further support our earlier findings of phenotypic differences between C. coli clade 2 and 3 isolates but also suggest that clade 2 and 3 isolates may differ in their virulence. However, more studies are needed to verify these *in vitro* findings.

## MATERIALS AND METHODS

### Bacterial isolates.

All C. coli clinical isolates (44, 52) and clade 2 and 3 water isolates (38) have been described previously (see the VA and F isolates in Table S1 in the supplemental material). The C. coli clade 1 LMG 6440 reference strain was also included for comparison.

### Bacterial culture conditions.

Bacteria were grown microaerobically (Oxoid Campygen; Thermo Fisher Scientific, Waltham, MA, USA) at 42°C and routinely resuscitated from frozen stocks by first growing them overnight on blood agar (Columbia agar plates supplemented with 5% horse blood; Oxoid, Basingstoke, UK), followed by overnight incubation in brucella broth (Becton, Dickinson and Company, Franklin Lakes, NJ, USA). To collect bacteria and/or broth, cultures were centrifuged at 8,000 × g for 5 to 10 min. Where indicated, the bacteria were heat inactivated at 70°C for 10 min, and complete inactivation was verified by plating on blood agar.

### Cell culture conditions.

The HT-29 human colon cancer cell line (ECACC 91072201) was maintained in RPMI 1640 medium (Gibco by Life Technologies, Carlsbad, CA, USA) supplemented with 2 mM glutamine (Swedish National Veterinary Institute, Uppsala, Sweden), 10% fetal bovine serum (FBS; Gibco by Life Technologies, Carlsbad, USA), 100 U/ml penicillin, and 100 µg/ml streptomycin (both from Swedish National Veterinary Institute, Uppsala, Sweden).

### *In vitro* cell adhesion assay.

Overnight bacterial cultures were centrifuged, diluted in cell culture medium, and added to low-passage HT-29 cells grown in RPMI 1640 medium supplemented with 2 mM glutamine and 1% FBS at a multiplicity of infection (MOI) of 100. At the time points indicated above, cells were photographed through a microscope lens and medium was collected for use in the IL-8 enzyme-linked immunosorbent assay (ELISA) and lactate dehydrogenase (LDH) assay. Cells were washed four times in phosphate-buffered saline (PBS) to remove nonadhered bacteria and lysed in 20 mM Tris, pH 7.5, 150 mM NaCl, 0.15% Triton X-100. The lysate was diluted 10 and 100 times for 16S rRNA qPCR analysis, which was also performed with starting cultures diluted 100 and 10,000 times, to determine the percent adhesion (bacteria collected in the lysate after infection/bacteria in the starting inoculum before infection). Mock-infected cells were used as a negative control in the qPCR analyses. Data from two to three independent infections were used, and two qPCRs were run on each sample. Results are presented as the mean and standard deviation for the isolates in each clade (clade 1, *n* = 3 isolates; clade 2, *n* = 5 isolates; clade 3, *n* = 4 isolates).

### IL-8 ELISA.

IL-8 levels in the medium were measured using an IL-8 ELISA kit (Thermo Fisher Scientific, Waltham, MA, USA) according to the manufacturer’s instructions. The medium was diluted 4 to 10 times prior to the assay. A standard of known concentration (included in the kit) was used to assess variations between infections. Results were expressed as the fold increase over the value for uninfected (mock-infected) cells. Data from two to three independent infections were used. The results are presented as the mean and standard deviation for the isolates in each clade (clade 1, *n* = 3 isolates; clade 2, *n* = 5 isolates; clade 3, *n* = 4 isolates).

### LDH assay.

The extracellular lactate dehydrogenase (LDH) activity in the cell culture medium was determined using a Pierce LDH cytotoxicity assay kit (Thermo Fisher Scientific, Waltham, MA, USA) according to the manufacturer’s protocol. Results were expressed as the relative fraction of healthy cells, with the value for uninfected (mock-infected) cells being set to 100%. Data from two to three independent infections were used. Results are presented as the mean and standard deviation for the isolates in each clade (clade 1, *n* = 3 isolates; clade 2, *n* = 5 isolates; clade 3, *n* = 4 isolates).

### RNA preparation from bacteria and cDNA synthesis.

Bacterial RNA was extracted from overnight cultures using an Isolate II RNA minikit (Bioline Reagents Ltd., London, UK) according to the manufacturer’s protocol. DNase I treatment (Ambion by Life Technologies, Carlsbad, CA, USA) was performed both on column and on the final eluted RNA. The concentration of the RNA was determined using a NanoDrop spectrophotometer, and the integrity of the RNA was verified on a 1% agarose gel. One microgram of RNA was reverse transcribed using a Maxima first-strand cDNA synthesis kit for qPCR (Thermo Fisher Scientific, Waltham, MA, USA) according to the manufacturer’s protocol. A control reaction without reverse transcriptase was set up for each sample to rule out residual DNA. A 1/2,000 fraction of the cDNA synthesis reaction mixture was used for qPCR. At least two independent RNA preparations were used for real-time qPCR.

### Quantitative PCR.

Real-time qPCR was performed in a Bio-Rad CFX96 Touch cycler using a DyNAmo HS SYBR green qPCR kit (Thermo Fisher Scientific, Waltham, MA, USA). The primer sequences for C. coli 16S rRNA and virulence genes are available upon request. For virulence gene expression analyses, the expression levels of each gene were first normalized to the expression level of 16S rRNA for each sample and run and thereafter expressed as the fold change over the level for the LMG 6440 reference strain. At least two independent qPCRs were run on each sample.

### WGS.

The DNA was extracted from overnight bacterial cultures of C. coli isolates F3, F4, F8, and LMG 6440 using a MagNA Pure compact nucleic acid isolation kit I (Roche, Penzberg, Germany) according to the manufacturer’s protocol (version 12). Whole-genome sequencing (WGS) was performed at the National Veterinary Institute of Sweden. The libraries for WGS were prepared with a Nextera XT sample preparation kit (Illumina, San Diego, CA, USA). An Illumina MiSeq platform with a 2 × 300 paired-end run was used for whole-genome sequencing. The single reads were assembled to contigs with the Velvet (version 7.0.4) program (53) running as a plug-in in Geneious (version 8.1.9) software (54).

Sequencing of clade 2 and 3 water isolates (38) and clinical clade 3 isolate 76339 (44) has been described previously. The whole-genome sequences of selected C. coli clade 1, 2, and 3 isolates (Table S1) were retrieved from the NCBI database (last accessed 10 September 2018).

### Genomics.

All *in silico* analyses of nucleotide and protein sequences were done on all 91 isolates listed in Table S1. The clade assignments of all isolates included were confirmed by average nucleotide identity (ANI)-based clustering of the whole genomes (55), followed by phylogenetic tree construction in SplitsTree4 (version 4.14.4) software (56). For virulence genes, ORF translations, sequence alignments, and neighbor-joining phylogenetic tree construction were done in CLC Main Workbench software (Qiagen, Hilden, Germany) using standard settings. No sequences with ambiguous nucleotides were used in the translation of the included genes. Calculations of sequence identities and clade-specific sequence variations were done manually.

### Statistics.

To identify statistically significant differences between clade 1 (*n* = 3), clade 2 (*n* = 5), and clade 3 (*n* = 4) groups, Student's *t* test (unpaired, two-tailed) was used. The level of significance is indicated in the figures and figure legends.

### Accession number(s).

GenBank accession numbers for isolates sequenced in this study are QYUT01000000 for F3, QYUS01000000 for F4, QYUU01000000 for F8, and QZCI01000000 for LMG 6440. All whole-genome sequences are available in GenBank, National Center for Biotechnology Information (https://www.ncbi.nlm.nih.gov/GenBank/index.html), under the accession numbers shown in Table S1.

## Supplementary Material

Supplemental file 1
