# Supplementary material for: Campylobacter coli Clade 3 Isolates Induce Rapid Cell Death In Vitro
Source: Appl Environ Microbiol. 2019 Feb 20;85(5):e02993-18. doi: 10.1128/AEM.02993-18 (PMC6384112; doi:10.1128/AEM.02993-18)
Supplement: Supplemental file 1 [file AEM.02993-18-s0001.pdf]

SUPPLEMENTAL MATERIAL

Campylobacter coli clade 3 isolates induce rapid cell death *in vitro*

Cecilia Johansson, Anna Nilsson, René Kaden, and Hilpi Rautelin

TABLE S1. *C. coli* clade 1 (n=11), clade 2 (n=24) and clade 3 (n=56) isolates included in genomic analyses.

Clade	Isolate	NCBI accession number	Source	Reference
1	BIGS0002	ANGM01000000	Clinical	(1)
1	BIGS0005	ANGP01000000	Pig feces	(1)
1	BIGS0015	ANGZ01000000	Pig feces	(1)
1	BIGS0017	ANHB01000000	Chicken meat	(1)
1	BIGS0019	ANHD01000000	Clinical	(1)
1	BIGS0021	ANHF01000000	Chicken meat	(1)
1	F3	QYUT01000000	Clinical	This study, (2)
1	F4	QYUS01000000	Clinical	This study, (2)
1	F8	QYUU01000000	Clinical	This study, (2)
1	LMG 6440 (NTCT11366)	QZCI01000000	Pig faeces Reference strain	This study
1	RM2228	AAFL01000000	Chicken carcass, Reference strain	(3)
2	A12B2	FBGG01000000	Environmental waters	
2	B15B3	FBFU01000000	Environmental waters	
2	BIGS0012	ANGW01000000	Clinical	(1)
2	F14B4C4	FBGM01000000	Environmental waters	
2	F15D2	FBFW01000000	Environmental waters	
2	H043880653	FBNC01000000	Clinical	
2	H054340287	FBOJ01000000	Clinical	
2	H055260513	FBNY01000000	Environmental waters	

2	H055260517	FAXG01000000	Environmental waters	
2	H091320798	FBKV01000000	Environmental waters	
2	H091940890	FBOS01000000	Environmental waters	
2	H092560131	FBKJ01000000	Environmental waters	
2	H093580632	FBPP01000000	Environmental waters	
2	H094560718	FBKY01000000	Environmental waters	
2	H105160209	FBQJ01000000	Environmental waters	
2	K11	FBGA01000000	Environmental waters	
2	M2D2	FBGB01000000	Environmental waters	
2	M4A4	FBGH01000000	Environmental waters	
2	M5D4	FBQV01000000	Environmental waters	
2	VA6	MPIQ01000000	Environmental waters	(4)
2	VA8	MPIS01000000	Environmental waters	(4)
2	VA24	MPIU01000000	Environmental waters	(4)
2	VA37	MPIV01000000	Environmental waters	(4)
2	VA46	MPIX01000000	Environmental waters	(4)
3	BIGS0003	ANGN01000000	Environmental waters	(1)
3	BIGS0009	ANGT01000000	Clinical	(1)
3	C9A1	FBQZ01000000	Environmental waters	
3	D13A4	FBFX01000000	Environmental waters	
3	H042280284	FBQS01000000	Clinical	
3	H042840283	FBPA01000000	Clinical	
3	H043560441	FBOR01000000	Clinical	
3	H051960469	FBOD01000000	Clinical	
3	H053120426	FBMY01000000	Environmental waters	

3	H060740717	FBIP01000000	Environmental waters
3	H060960756	FBGE01000000	Environmental waters
3	H060960757	FBHG01000000	Environmental waters
3	H061680633	FBOE01000000	Environmental waters
3	H061980521b	FBNK01000000	Environmental waters
3	H063720401	FBPO01000000	Clinical
3	H065100506	FBKP01000000	Environmental waters
3	H065160534	FBJV01000000	Environmental waters
3	H070380306	FBKT01000000	Clinical
3	H071960681	FBNO01000000	Environmental waters
3	H072620566	FBKO01000000	Environmental waters
3	H072760517	FBQO01000000	Clinical
3	H073180383	FAZM01000000	Clinical
3	H073220518	FBNQ01000000	Environmental waters
3	H073580401	FBAL01000000	Environmental waters
3	H074080509	FBOA01000000	Environmental waters
3	H074080511	FBNP01000000	Environmental waters
3	H075140555	FBKA01000000	Environmental waters
3	H075200522	FBMZ01000000	Environmental waters
3	H081380695a	FBNU01000000	Environmental waters
3	H081840386	FBLJ01000000	Clinical
3	H082280513	FAZO01000000	Environmental waters
3	H082280515	FAZW01000000	Environmental waters
3	H082560569	FAYV01000000	Environmental waters
3	H083420694	FBNG01000000	Environmental waters

3	H083420701	FBNT01000000	Environmental waters	
3	H085160742	FBJZ01000000	Environmental waters	
3	H085160749	FBLD01000000	Environmental waters	
3	H090980249	FBKL01000000	Environmental waters	
3	H091000385	FBNE01000000	Clinical	
3	H092260569b	FBNM01000000	Environmental waters	
3	H093580324	FBKN01000000	Environmental waters	
3	H094560713	FBKD01000000	Environmental waters	
3	H094560720	FBKQ01000000	Environmental waters	
3	H094860392	FBAT01000000	Environmental waters	
3	H102740169	FBPR01000000	Environmental waters	
3	H110340458	FBQC01000000	Environmental waters	
3	H113760461	FBOH01000000	Clinical	
3	H124500577	FBOV01000000	Clinical	
3	H124620276a	FBOI01000000	Clinical	
3	H132840800	FBPJ01000000	Environmental waters	
3	H133380250	FBQM01000000	Clinical	
3	K3D1	FBFT01000000	Environmental waters	
3	VA7	MPIR01000000	Environmental waters	(4)
3	VA15	MPIT01000000	Environmental waters	(4)
3	VA38	MPIW01000000	Environmental waters	(4)
3	76339	HG326877	Clinical	(5)

SUPPLEMENTAL REFERENCES

1. Sheppard SK, Didelot X, Jolley KA, Darling AE, Pascoe B, Meric G, Kelly DJ, Cody A, Colles FM, Strachan NJ, Ogden ID, Forbes K, French NP, Carter P, Miller WG, McCarthy ND, Owen R, Litrup E, Egholm M, Affourtit JP, Bentley SD, Parkhill J, Maiden MC, Falush D. 2013. Progressive genome-wide introgression in agricultural Campylobacter coli. Mol Ecol 22:1051-64.

2. Feodoroff FB, Lauhio AR, Sarna SJ, Hanninen ML, Rautelin HI. 2009. Severe diarrhoea caused by highly ciprofloxacin-susceptible Campylobacter isolates. Clin Microbiol Infect 15:188-92.

3. Fouts DE, Mongodin EF, Mandrell RE, Miller WG, Rasko DA, Ravel J, Brinkac LM, DeBoy RT, Parker CT, Daugherty SC, Dodson RJ, Durkin AS, Madupu R, Sullivan SA, Shetty JU, Ayodeji MA, Shvartsbeyn A, Schatz MC, Badger JH, Fraser CM, Nelson KE. 2005. Major structural differences and novel potential virulence mechanisms from the genomes of multiple campylobacter species. PLoS Biol 3:e15.

4. Nilsson A, Skarp A, Johansson C, Kaden R, Engstrand L, Rautelin H. 2018. Characterization of Swedish Campylobacter coli clade 2 and clade 3 water isolates. Microbiologyopen doi:10.1002/mbo3.583.

5. Skarp-de Haan CPA, Culebro A, Schott T, Revez J, Schweda EK, Hänninen ML, Rossi M. 2014. Comparative genomics of unintrogressed Campylobacter coli clades 2 and 3. BMC Genomics 15:129.

SUPPLEMENTAL FIGURE LEGENDS

Figure S1. Neighbour-joining tree based on protein sequence alignments of CadF from *C. coli* clade 1, 2 and 3 isolates (Table S1) together with the *C. jejuni* reference strains 11168 and 81-176. Node colours indicate clades; Clade 1 – blue, Clade 2 – green and Clade 3 – red. The exact positions of the isolates in this study are shown. Scale bar represent 0.01.

Figure S2. Alignment of CadF protein sequences from *C. coli* clade 1, 2 and 3 isolates (Table S1) together with the *C. jejuni* reference strains 11168 and 81-176. Dash; amino acid is missing, grey box; site with variation, asterisk; site where clade 3 isolates differ from clade 1 and 2 isolates, bold; site where clade 3 is identical to *C. jejuni*, box; conserved fibronectin binding site, underlined sequence; OmpA-like domain (amino acids 167-324).

Figure S3. Neighbour-joining tree based on protein sequence alignments of CeuE from *C. coli* clade 1, 2 and 3 isolates (Table S1) together with the *C. jejuni* reference strains 11168 and 81-176. Node colours indicate clades; Clade 1 – blue, Clade 2 – green and Clade 3 – red. The exact positions of the isolates in this study are shown. Scale bar represent 0.01.

Figure S1.

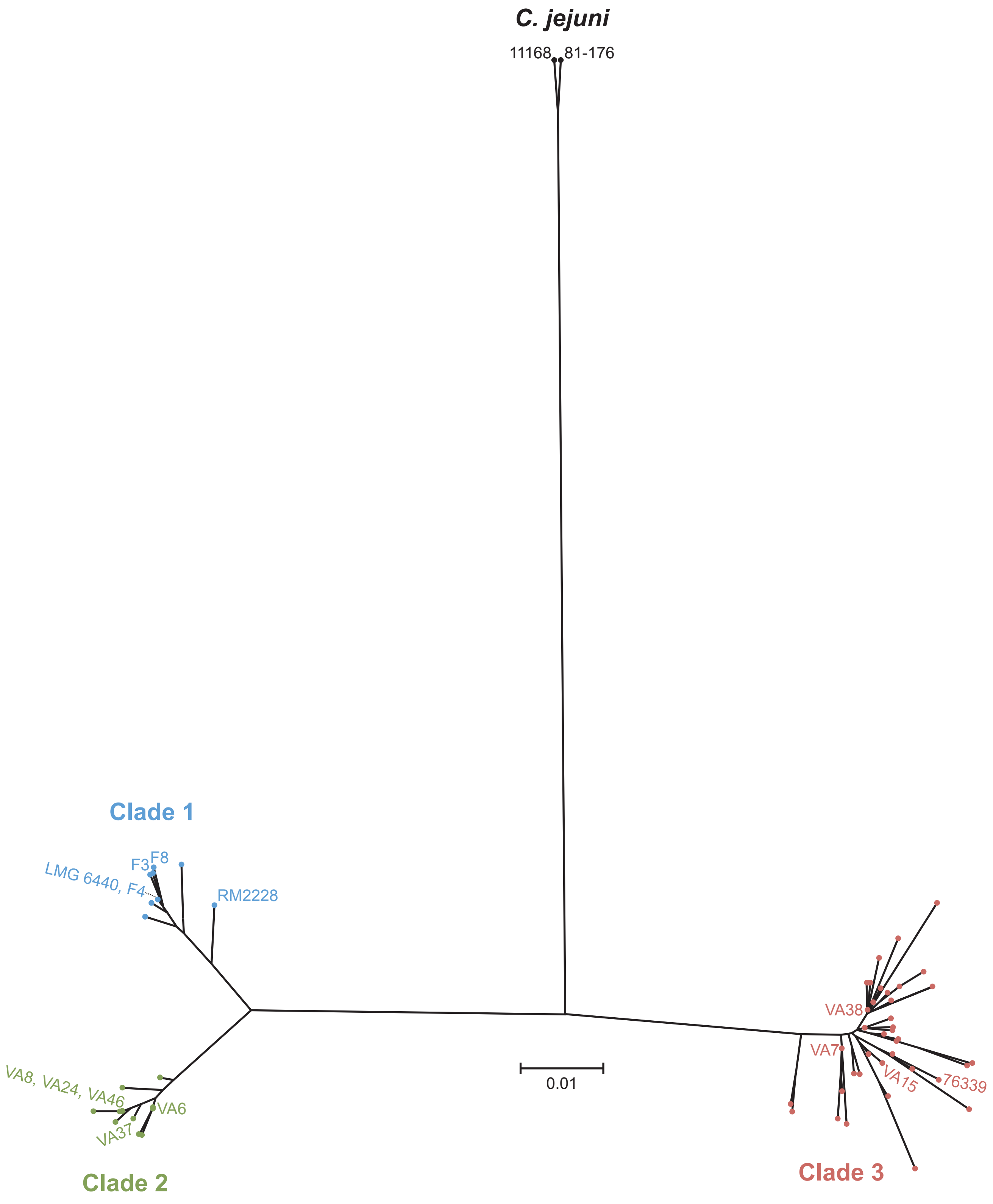

Figure S1. Neighbour-joining tree based on protein sequence alignments of CadF from *C. coli* clade 1, 2 and 3 isolates (Table S1) together with the *C. jejuni* reference strains 11168 and 81-176. Node colours indicate clades; Clade 1 – blue, Clade 2 – green and Clade 3 – red. The exact positions of the isolates in this study are shown. Scale bar represent 0.01.

Figure S2.

```
                          10         20         30         40         50
                           |          |          |          |          |
                   *             *                                   *
C. coli clade 1   MRKLLLCLGL SSVLFGADNN VKFEITPTLN YNYFEGNLDM DNRYAPGIRL
C. coli clade 2   MRKLLLCLGL ASVLFGADNN VKFEITPTLN YNYFEGNLDM DNRYAPGIRL
C. coli clade 3   MKKLLLCLGL ASVLLGADNN VKFEITPTLN YNYFEGNLDM DNRYAPGVRL
C. jejuni 11168   MKKIFLCLGL ASVLFGADNN VKFEITPTLN YNYFEGNLDM DNRYAPGIRL
C. jejuni 81-176  MKKILLCLGL ASVLFSADNN VKFEITPTLN YNYFEGNLDM DNRYAPGIRL

C. coli clade 1   GYHFDDFWLD QLELGLEHYS DVKYTNSTLT TDITRTYLSA IKGIDLGEKF
C. coli clade 2   GYHFDDFWLD QLELGLEHYS DVKYTNSTLT TDITRTYLSA IKGIDLGEKF
C. coli clade 3   GYHFDDFWLD QLELGLEHYS DVKYTNSTLT TDITRTYLSA IKGIDLGEKF
C. jejuni 11168   GYHFDDFWLD QLEFGLEHYS DVKYTNTNKT TDITRTYLSA IKGIDVGEKF
C. jejuni 81-176  GYHFDDFWLD QLEFGLEHYS DVKYTNTNKT TDITRTYLSA IKGIDVGEKF

C. coli clade 1   YFYGLAGGGY EDFSKGAFDN KSGGFGHYGA GLKFRLSDSL ALRLETRDQI
C. coli clade 2   YFYGLAGGGY EDFSHGAFDN KSGGFGHYGA GLKFRLSDSL ALRLETRDQI
C. coli clade 3   YFYGLAGGGY EDFSKGAFDN KSGGFGHYGA GLKFRLSDSL ALRLETRDQI
C. jejuni 11168   YFYGLAGGGY EDFSNAAYDN KSGGFGHYGA GVKFRLSDSL ALRLETRDQI
C. jejuni 81-176  YFYGLAGGGY EDFSNAAYDN KSGGFGHYGA GVKFRLSDSL ALRLETRDQI

                     * *                  *    *   *   * * *  ** *******
C. coli clade 1   SFHDADHSWV STLGISFGFG AKQEKVVVEQ TKEVVNKPQV VTPAPAPVVS
C. coli clade 2   SFHDADHSWV STLGVSFGFG AKQKKVVVEQ TKEVVSKPQV VTPAPAPVVS
C. coli clade 3   SFHNANHSWV STLGISFGFG AKREKVVAEQ VKEVAIEPRV AAPV------
C. jejuni 11168   NFNHANHNWV STLGISFGFG GKKEKAV-E- --EVADT-R- ATP-------
C. jejuni 81-176  NFNHANHNWV STLGISFGFG GKKEKAV-E- --EVADT-R- ATP-------

                    *          *           **    *     *   *
C. coli clade 1   QSKCPEEPRE GALLDENGCE KTIYLEGHFD FDKVNINPAF EEQIKEIAQI
C. coli clade 2   QSKCPAEPRE GALLDENGCE KTIYLEGHFD FDKVNINPAF EEKIKEIAKI
C. coli clade 3   QSQCPAEPRE GAMLDENGCE KTISFEGHFG FDKVDINPVF EEKIKEIAQI
C. jejuni 11168   QAKCPVEPRE GALLDENGCE KTISLEGHFG FDKTTINPTF QEKIKEIAKV
C. jejuni 81-176  QAKCPVEPRE GALLDENGCE KTISLEGHFG FDKTTINPTF QEKIKEIAKV

                      *                  *                          *
C. coli clade 1   LDENVRYDTI LEGHTDNIGS RSYNQKLSER RANSVAKELE KFGVDKSRIQ
C. coli clade 2   LDENVRYDTI LEGHTDNTGS RSYNQKLSER RAESVAKELE KFGVDKSRIQ
C. coli clade 3   LDENARYDTI LEGHTDNIGS RAYNQKLSER RAESVAKELE KFGVDKDRIQ
C. jejuni 11168   LDENERYDTI LEGHTDNIGS RAYNQKLSER RAKSVANELE KYGVEKSRIK
C. jejuni 81-176  LDENERYDTI LEGHTDNIGS RAYNQKLSER RAKSVANELE KYGVEKSRIK

                                             *      *
C. coli clade 1   TVGYGQDKPR SSNDTKEGRA DNRRVEAKFI LN
C. coli clade 2   TVGYGQDKPR SSNDTKEGRA DNRRVEAKFI LN
C. coli clade 3   TVGYGQDKPR SSNDTKEGRA DNRRVDAKFI LR
C. jejuni 11168   TVGYGQDNPR SSNDTKEGRA DNRRVDAKFI LR
C. jejuni 81-176  TVGYGQDNPR SSNDTKEGRA DNRRVDAKFI LR
```

Figure S2. Alignment of CadF protein sequences from *C. coli* clade 1, 2 and 3 isolates (Table S1) together with the *C. jejuni* reference strains 11168 and 81-176. Dash; amino acid is missing, grey box; site with variation, asterisk; site where clade 3 isolates differ from clade 1 and 2 isolates, bold; site where clade 3 is identical to *C. jejuni*, box; conserved fibronectin binding site, underlined sequence; OmpA-like domain (amino acids 167-324).

Figure S3.

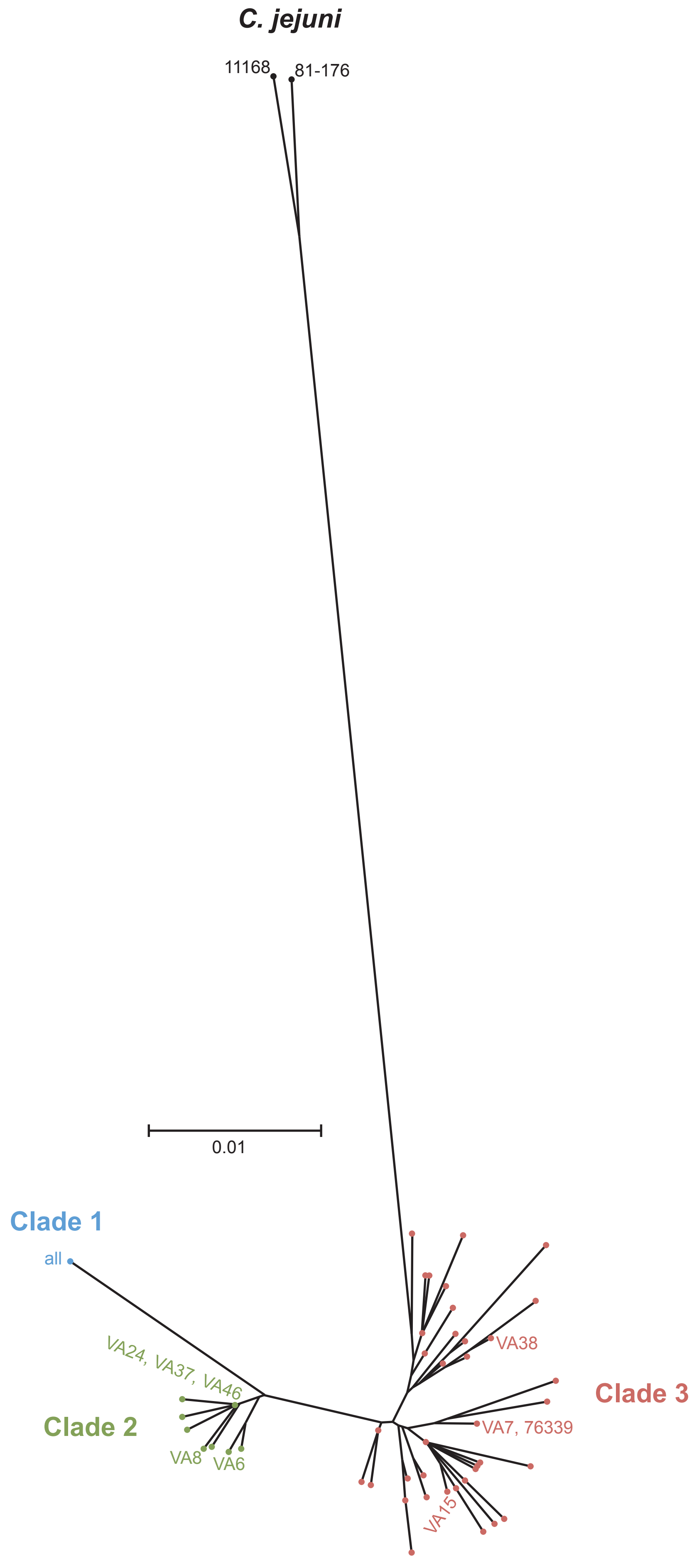

Figure S3. Neighbour-joining tree based on protein sequence alignments of CeuE from *C. coli* clade 1, 2 and 3 isolates (Table S1) together with the *C. jejuni* reference strains 11168 and 81-176. Node colours indicate clades; Clade 1 – blue, Clade 2 – green and Clade 3 – red. The exact positions of the isolates in this study are shown. Scale bar represent 0.01.
